# Prognostic Value of Concomitant Bronchiectasis in Newly Diagnosed Diffuse Panbronchiolitis Patients on a Maintenance Therapy with Macrolides

**DOI:** 10.1155/2019/4913814

**Published:** 2019-03-10

**Authors:** Benyong Xu, Yanhua Mao, Xiaoyu Wan, Jianhui Chen, Meiping Ye, Mengling Zhan, Liyun Xu, Lan Zhao, Bing Li, Zhemin Zhang, Yang Liu, Haiqing Chu

**Affiliations:** ^1^Tongji University School of Medicine, Shanghai 200092, China; ^2^Department of Respiratory Medicine, Shanghai Pulmonary Hospital, Tongji University School of Medicine, Shanghai 200433, China; ^3^Institute of Antibiotics, Huashan Hospital, Fudan University, Shanghai 200040, China; ^4^Shanghai Key Laboratory of Tuberculosis, Shanghai Pulmonary Hospital, Tongji University School of Medicine, Shanghai 200433, China

## Abstract

**Background:**

Factors determining the prognosis of diffuse panbronchiolitis (DPB) remain unclear at present. The objective of this study was to identify the prognostic value of concomitant bronchiectasis in the macrolide treatment efficacy and exacerbation risk in DPB patients.

**Methods:**

Data of patients initially diagnosed with DPB at the Shanghai Pulmonary Hospital between January 2007 and December 2017 were retrospectively collected and analyzed. The patients were divided into two groups according to the existence of bronchiectasis. Clinical manifestations, laboratory findings, microbiological culture results, as well as exacerbation risks and treatment outcomes, were compared between these two groups. The survival curve and Cox regression analysis models were additionally constructed to further demonstrate the predicting role of bronchiectasis in DPB exacerbation.

**Results:**

Baseline data revealed more respiratory symptoms, lower body mass index (BMI), and forced expiratory volume in one second (FEV_1_) as well as increased isolates of *Pseudomonas aeruginosa* (*P. aeruginosa*) in DPB subjects with bronchiectasis than those without. Furthermore, bronchiectasis was associated with a lower rate of responsiveness to macrolides and increased exacerbation frequency during follow-up. The survival curve and Cox regression analysis showed that comorbid bronchiectasis was linked to increased time to episode relapse, which remained significant even after controlling for BMI, FEV_1_, and *P. aeruginosa* culture results.

**Conclusion:**

The coexistence of bronchiectasis predicted a poor outcome of maintenance macrolide therapy and an increased exacerbation risk in DPB subjects, possibly through its impacts on nutritional status, pulmonary function, and *P. aeruginosa* infections.

## 1. Introduction

Diffuse panbronchiolitis (DPB) is an idiopathic inflammatory disease, which affects the distal airways and predominantly the transition zone between the respiratory bronchioles and alveoli [1]. It is characterized by progressive suppurative and obstructive airway disease. If left untreated, DPB will progress to bronchiectasis, respiratory failure, and eventually death [2]. The prognosis of DPB was poor before the long-term treatment with erythromycin was introduced. Since then, the 10-year survival rate has improved from 33.2% to 90% [3]. However, there are still cases in which the benefit is minimal and the disease relapses or progresses in spite of causes of macrolide antibiotics treatment. The factors that affect treatment efficacy and relapse are still unknown [4, 5].

Bronchiectasis is a chronic lung disease characterized by a vicious cycle of airway infection and inflammation, leading to permanent structural damage to the small airways and sometimes to the surrounding lung parenchyma [6]. The clinical presentation can overlap with other respiratory disorders such as chronic obstructive pulmonary disease (COPD), asthma, obstructive sleep apnea-hypopnea syndrome (OSAHS), and so on, forming overlap syndromes [7–9]. Recently, several reports indicated that comorbid bronchiectasis was associated with poorer treatment efficacy and prognosis for various other chronic lung diseases. Bronchiectasis comorbidity also resulted in increased frequency of cases with difficult-to-treat pathogenic bacteria, decreased lung function, and increased risk of death from all causes, among other symptoms [10, 11]. DPB has a very well-established relationship with bronchiectasis. DPB can occur in conjunction with bronchiectasis or progress to severe bronchiectasis in its advanced stage. Bronchiectasis in conjunction with DPB has been assumed a more severe syndrome in DPB. However, this conclusion was mainly based on clinical observations and expert opinions. Confirmation and detailed data from systematic clinical research are still lacking.

We hypothesized that bronchiectasis would be a negative factor for DPB treatment and exacerbation risk, similar to its role in other chronic lung diseases. Therefore, we conducted a retrospective cohort study to identify the prognostic value of bronchiectasis in DPB, with the underlying mechanisms explored.

## 2. Patients and Methods

### 2.1. Patients and Data Collection

**Figure 1:**
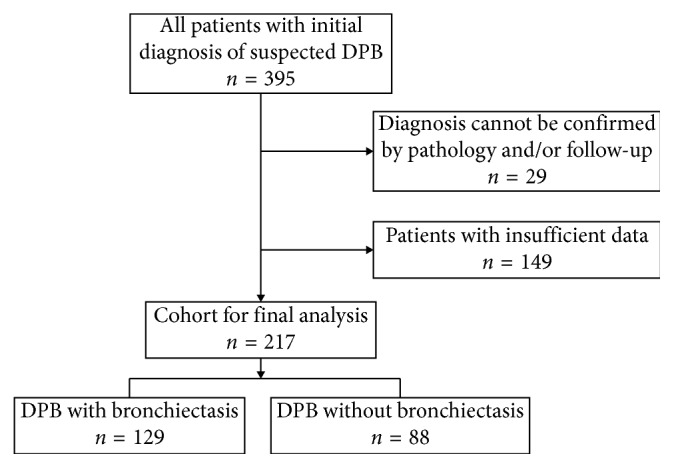
Flow chart for selecting the cohort of DPB patients.

Data of 395 cases with initially suspected DPB from 2007 to 2017, who were then prescribed a macrolide regimen and asked for follow-up on a regular basis, were retrospectively screened and collected from the Shanghai Pulmonary Hospital. Among them, 217 were finally enrolled into the cohort, following the flow chart for selecting the eligible patients that is shown in Figure 1. All patients were well documented with detailed clinical information, including baseline demographic data, arterial blood gas analysis, blood cold hemagglutination (CHA) testing, pulmonary function tests, and microbiological and laboratory results of sputum and bronchial lavage fluid. The radiological findings were also required, with all patients presented typical bilateral diffused tree-in-bud patterns. Lung biopsy was performed in 10 cases, all of which were pathologically consistent with DPB. During the follow-up period, information on treatment efficacy and exacerbation frequency was collected and assessed. Patients were divided into two groups according to the existence of bronchiectasis in chest CT scan images, except for which no meaningful differences were found. The study was approved by the Ethical Committee of Tongji University, Shanghai Pulmonary Hospital. All of the participants signed informed consent for any procedures that were relevant to this study.

### 2.2. Diagnostic Criteria of DPB and Bronchiectasis

The DPB diagnostic criteria were in accordance with the guidelines proposed by a working group of the Ministry of Health and Welfare of Japan [12]. A chest high-resolution computed tomography (HRCT) scan was used to confirm the diagnosis of bronchiectasis, based on the diagnostic criteria proposed by Naidich et al. [13]. Cases with small bronchiectasis that was only visible in a single pulmonary segment, which also can be detected in healthy people, were excluded. Patients with bronchiectasis caused by congenital diseases such as cystic fibrosis and Marfan syndrome were not found in this study.

### 2.3. Assessment of Treatment Efficacy

The treatment strategy was a combination of macrolide antibiotics and other measures, including glucocorticoids, antibiotics against infectious pathogens, oxygen therapy, symptomatic and supportive therapy, etc [2, 12, 14]. The patient's response to macrolide antibiotics treatment was defined as follows [15]: (1) Healed: all symptoms and abnormal body signs disappeared; chest CT, arterial blood gas analysis, and lung function returned to normal. (2) Improved: more than one of the clinical symptoms, body signs, or imaging scan improved after treatment. (3) Unchanged: clinical symptoms and abnormal body signs persisted with no improvement observed in the chest CT. (4) Deteriorating: one or more clinical symptoms, abnormal body signs, or chest CT were aggravated/became worse. (5) Recurrent: healed after treatment, but clinical or radiological regression occurred during follow-up. Conditions (1) or (2) was considered as responsive. Conditions (3), (4), and (5) were considered as unresponsive. The symptoms and body signs were documented in the medical records. CT images were analyzed and interpreted by two radiologists and a pulmonologist who were blinded to the case. The medical records and radiological reports were evaluated by two pulmonologists to confirm the accuracy of the extracted information.

### 2.4. Definition of DPB Exacerbations

An exacerbation of DPB was defined, in our study, as the presence for at least 24 h of increased symptoms (including cough, sputum volume and/or consistency, sputum purulence, breathlessness and/or exercise tolerance, fatigue and/or malaise, hemoptysis), with or without an increase in tree-in-bud in CT scans, resulting in an unscheduled clinical visit and a subsequent hospital admission.

### 2.5. Statistical Analysis

Statistical analysis was carried using SPSS version 20 (IBM Corporation, Chicago, IL, USA). Group comparisons for continuous data were performed using either Student's *t*-test (normally distributed data) or Mann–Whitney *U* test (nonnormally distributed data). Group comparisons of proportions were made using Pearson's chi-squared test or Fisher's exact test. The survival curve of the time to exacerbation for the two groups (i.e., DPB patients with and without bronchiectasis) was constructed according to the Kaplan–Meier method and was compared using the log-rank test. A multivariate stepwise Cox regression analysis was performed until the most parsimonious model was achieved to assess the association between the concomitant bronchiectasis and the time to exacerbation after initial DPB diagnosis and beginning of macrolides treatment, adjusting for age, gender, BMI, smoking status, disease duration, chronic sinusitis, CHA test, FEV_1_, and respiratory tract cultures for *P. aeruginosa*. The confounders were either of clinical interest or were selected from the demographic, clinical, and experimental findings as well as therapeutic regimens, listed in Tables 1 and 2, through a univariate Cox regression analysis, with factors with a *P* value of <0.1 entering into the final analysis. *P* values of <0.05 were considered statistically significant.

## 3. Results

### 3.1. Baseline Demographic, Clinical, and Laboratory Findings between DPB Patients with and without Bronchiectasis

A total of 217 from 395 DPB patients were included into the final analysis. Bronchiectasis was present in 129 patients. The baseline characteristics for all patients are shown in Table 1. DPB patients with bronchiectasis demonstrated significantly lower BMI than those without bronchiectasis (*P* < 0.001). No significant difference in age, sex, smoking status, personal history, and previous disease history existed between the two groups.

There was a weak trend towards a longer DPB duration in patients with bronchiectasis (*P*=0.088). Cough (100%), sputum production (95%), exertional dyspnea (89.4%), and shortness of breath (76.5%) were the most common symptoms in each group, followed by hemoptysis (23.0%) and body weight loss (15.2%). More patients in the bronchiectasis group suffered from short breath (*P* < 0.001), hemoptysis (*P*=0.039), and body weight loss (*P*=0.038).

Arterial blood gas analysis showed a lower level of arterial partial pressure of oxygen (P_a_O_2_) in DPB subjects with bronchiectasis (*P*=0.037). No significant differences in blood CHA test, white blood cell count, neutrophil percentages, C-reactive protein (CRP), or erythrocyte sedimentation rate (ESR) were found between the two groups.

Both groups exhibited damaged pulmonary functions, which were more pronounced in the DPB subjects with bronchiectasis, as reflected by lower levels of forced expiratory volume in one second (FEV_1_, *P*=0.040), forced vital capacity (FVC, *P*=0.058), and *FEV*_1_/*FVC* (*P*=0.070), with the former reaching statistical significance. No difference in forced expiratory flow 75% (FEF75) and residual volume (RV) was found.

*Pseudomonas aeruginosa* (*P. aeruginosa*) was the most frequently detected pathogenic bacteria (35.5%) in all the subjects, followed by *Haemophilus influenzae* (15.7%), *Candida albicans* (8.8%), and *Neisseria bacteria* (7.4%). Only *P. aeruginosa* was more frequently isolated in DPB patients with bronchiectasis (*P* < 0.001).

### 3.2. Follow-Up Outcomes between DPB Patients with and without Bronchiectasis

Therapeutic regimens and efficacy assessments are shown in Table 2. There were no differences in the selection of macrolide antibiotics and regular use of other auxiliary medicines, including inhaled corticosteroid (ICS), long-acting bronchial agonist (LABA), and long-acting muscarinic antagonist (LAMA). DPB patients with bronchiectasis experienced more aggravating events (*P*=0.048). The rate of responsiveness (healed plus improved) was significantly lower in DPB patients with bronchiectasis than those without (*P* < 0.001).

**Figure 2:**
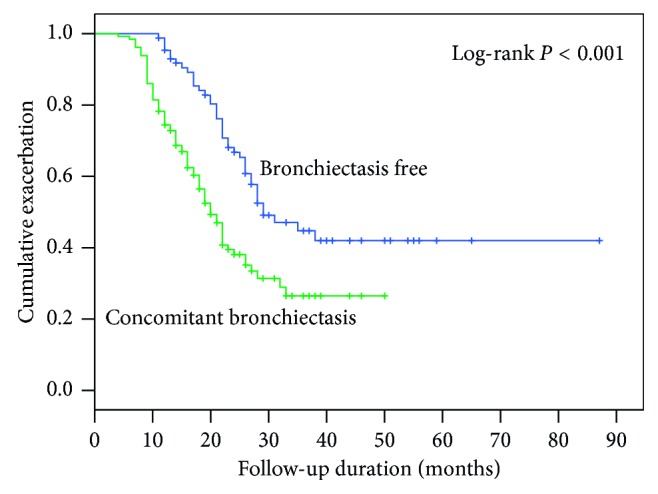
Kaplan–Meier survival curve and log-rank test were used to examine the association between the concomitant bronchiectasis in DPB subjects and the time to exacerbation since the beginning of maintenance therapy with macrolides. The results showed that bronchiectasis remarkably potentiated the risk of exacerbation events.

The Kaplan–Meier survival and log-rank test results showed a significant difference in the time to exacerbation after the beginning of macrolide therapy between patients with and without bronchiectasis (*P* < 0.001), with the estimated value of 20.0 (95% confidence interval (CI): 17.6–22.4) months and 29.0 (95% CI: 21.7–36.3) months, respectively (Figure 2). Further analysis via Cox regression analysis revealed bronchiectasis as an independent factor. The unadjusted odds ratio (OR) of 2.121 (95% CI: 1.437–3.131, *P* < 0.001) remained statistically significant after correction for confounders, including BMI, FEV_1_, and positive *P. aeruginosa* isolates (OR 1.545 (95% CI: 1.016–2.349, *P*=0.042)) (Table 3).

## 4. Discussion

In this paper, we conducted a relatively large retrospective cohort study, enrolling 217 subjects. To our knowledge, no other study in the literature has surpassed this case number. Our study also captured data from a long follow-up duration (up to 87 months) to confirm the potential adverse impact of comorbid bronchiectasis in newly diagnosed DPB patients on the efficacy of macrolide maintenance therapy and the exacerbation risk. As expected, our results demonstrated a poorer outcome of macrolide treatment, as well as a shorter period to episode relapse and increased exacerbation frequency since study enrollment in newly diagnosed DPB patients with bronchiectasis compared with their bronchiectasis-free counterparts. Meanwhile, bronchiectasis was also associated with accelerated chronic wasting, more symptoms, poorer lung function, and increased isolates of *P. aeruginosa* at baseline. According to the multiple Cox regression analysis, comorbid bronchiectasis (in combination with a couple of other clinically significant confounding variables including BMI, FEV_1_, and positive *P. aeruginosa* culture) was a strong independent factor of macrolide treatment. It could thus be speculated that bronchiectasis exerts a detrimental influence on DPB disease control and prognosis, at least through pathways involving nutritional condition, pulmonary function, and lower airway tract infections.

*P. aeruginosa* is a well-documented pathogen that provokes an intense inflammatory response leading to persistent airway inflammation and airway structural damage [16, 17]. It has been reported that in the early stage of DPB, *Haemophilus influenzae* represents the most common colonized microbiolgical agent (44%), followed by *P. aeruginosa* (22%). Colonization with *P. aeruginosa* eventually occurs, and the detection rate of *P. aeruginosa* in DPB can rise to 60%. This accelerates disease progression, causing a wide range of severe opportunistic infections [1]. Chronic colonization by *P. aeruginosa* is associated with a greater decline in pulmonary function and more frequent exacerbation and hospitalization as well as increased mortality [18, 19]. Consistently, in our study, occurrence of *P. aeruginosa* was more frequently detected in DPB patients with bronchiectasis and was partly responsible for the poor therapeutic outcomes.

Nutritional status is frequently underestimated due to a lack of awareness by health professionals who deal with different chronic respiratory diseases. However, nutritional abnormalities are highly prevalent in this field, leading to important clinical consequences [20]. It has been reported in COPD patients that those with malnutrition present higher degrees of airway obstruction, perception of dyspnea, and CAT scores while having lower exercise capacity [21, 22]. As for bronchiectasis, fat-free mass depletion is related to increased inflammatory activity [23]. There are no published data to our knowledge that refer to the relationship between DPB and malnutrition. Therefore, for the first time, our study presented evidence that lower BMI is linked to likelihood of comorbid bronchiectasis and as such has a negative impact in therapeutic outcome. This link justifies a nutritional intervention in the treatment of DPB. The underlying mechanisms are still unknown. However, it may involve impairment of immunological function and increased susceptibility to and severity of community-acquired pneumonia [24–26]. Weakness of the respiratory muscles may also play a role.

Pulmonary dysfunction could be a natural consequence of long-term chronic airway inflammation and recurrent infectious episodes. In our manuscript, a decrease in pulmonary function, reflected by a lower FEV_1_ value, represented a third confounding risk factor in addition to *P. aeruginosa* infection and malnutrition in predicting a shortened duration of DPB aggravation since maintenance macrolide therapy. This finding was consistent with the established role of FEV_1_ in the assessment and prognosis of asthma and COPD [27, 28]. Notably, no difference in FEF75 was documented in our reports, and in view of the large and small airway involvement of FEV_1_ and FEF75, respectively, our results suggested that coexistence of bronchiectasis affected large rather than small airways of DPB patients.

It should be pointed out that concomitant bronchiectasis remained significant in the final Cox regression model, even after adjusting for all of the abovementioned factors, indicating other pathways that are involved in the relationship between bronchiectasis and DPB but were not identified here. However, it is reasonable to assume that the irreversible airway damage, the persistent inflammatory response, and the resulting recurrent infections (features involved in bronchiectasis), which cannot be reversed even with an effective therapy, may help explain the difficulty in treating and the poor prognosis of DPB in combination with bronchiectasis.

Several issues and limitations should be mentioned here. First, bronchiectasis may be considered as a primary or secondary condition, which could not be distinguished during our data collection due to the retrospective nature of this study. Second, since there was a marginal (but not statistically significant) difference in DPB duration between our patients with and without bronchiectasis, it is thus possible that the poorer therapeutic effects and prognosis found in the bronchiectasis group might only represent the consequence of advanced or end-stage DPB. However, we do not think this is the case. By using a multiple Cox regression analysis to predict treatment responsiveness, DPB duration was finally excluded from the model, but bronchiectasis remained a predictor of treatment outcome. In addition, we cannot determine whether the *P. aeruginosa* was a colonizing or infecting bacteria. Furthermore, the virulence and resistance of the bacteria cannot be defined either, which will also affect the treatment efficacy and prognosis.

## Figures and Tables

**Table 1:** Demographic, clinical, and laboratory findings of DPB subjects with and without bronchiectasis at baseline.

Parameter	Whole group (*n*=217)	DPB with bronchiectasis (*n*=129)	DPB without bronchiectasis (*n*=88)	*P* value^▲^
Age (years)	52.5 ± 16.8	53.0 ± 16.6	51.9 ± 17.3	0.597
Female	91 (41.9)	59 (45.7)	32 (36.4)	0.108
BMI (kg/m^2^)	**21.3** **±** **5.3**	**19.7** **±** **3.9**	**23.8** **±** **6.2**	**<0.001**
Smoking history	52 (24.0)	34 (26.4)	18 (20.5)	0.317
Disease duration (year)^※^	7.0 (5.0, 9.0)	7.0 (5.0, 10.5)	6.5 (5.0, 9.0)	0.088
Disease history				
Chronic sinusitis	197 (90.8)	116 (89.9)	81 (92.0)	0.596
Chronic bronchitis	19 (8.8)	14 (10.9)	5 (5.7)	0.186
Emphysema	11 (5.1)	7 (5.4)	4 (4.5)	0.771
Preexisting tuberculosis	24 (11.1)	17 (13.2)	7 (8.0)	0.228
Hypertension	42 (19.4)	23 (17.8)	19 (21.6)	0.491
Diabetes	10 (4.6)	6 (4.7)	4 (4.5)	0.971
Cardiovascular diseases	14 (6.5)	9 (7.0)	5 (5.7)	0.703
Rheumatic diseases	10 (4.6)	6 (4.7)	4 (4.5)	0.971
Pulmonary surgery history	9 (4.1)	7 (5.4)	2 (2.3)	0.253
Clinical manifestations				
Cough	217 (100)	129 (100)	88 (100)	1.000
Sputum production	216 (99.5)	129 (100)	87 (98.9)	1.000
Shortness of breath	**166 (76.5)**	**112 (86.82)**	**54 (61.4)**	**<0.001**
Exertional dyspnea	194 (89.4)	118 (91.5)	76 (86.4)	0.230
Hemoptysis	**50 (23.0)**	**36 (27.9)**	**14 (15.9)**	**0.039**
Body weight loss	**33 (15.2)**	**25 (19.4)**	**8 (9.1)**	**0.038**
Crackle	119 (54.8)	72 (55.8)	47 (53.4)	0.727
Wheezing	53 (24.4)	30 (23.3)	23 (26.1)	0.628
Laboratory findings				
pH	7.41 (7.39, 7.43)	7.41 (7.39, 7.43)	7.41(7.39, 7.43)	0.674
P_a_CO_2_ (mmHg)	40.7 (37.2, 45.3)	40.8 (37.1, 45.6)	40.5 (38.0, 44.7)	0.865
P_a_O_2_ (mmHg)	**80.0** **±** **15.7**	**78.2** **±** **14.7**	**82.7** **±** **16.8**	**0.037**
WBC (10 × 9/L)	7.6 ± 2.7	7.3 ± 2.6	7.9 ± 2.7	0.143
Neutrophil (%)	63.3 ± 11.7	64.3 ± 11.4	61.9 ± 11.9	0.134
CRP (mg/L)	4.8 (1.6, 12.4)	4.6 (1.6, 15.0)	5.5 (1.5, 11.7)	0.687
ESR (mm/h)	20.2 (9.2, 36.7)	19.6 (8.0, 35.4)	20.4 (10.3, 37.4)	0.522
Positive CHA test^■^	49 (22.6)	30 (23.3)	19 (21.6)	0.773
Pulmonary function tests				
FVC (% pred)	74.8 ± 20.6	72.6 ± 20.9	78.0 ± 19.9	0.058
FEV_1_ (% pred)	**60.7** **±** **15.4**	**58.9** **±** **14.1**	**63.3** **±** **16.8**	**0.040**
FEV_1_/FVC	65.0 ± 8.1	64.1 ± 7.4	66.2 ± 8.9	0.070
FEF75 (% pred)	39.8 (32.6, 55.9)	38.2 (34.1, 54.5)	41.4 (29.3, 57.5)	0.712
RV (% pred)	149.4 (123.3, 178.5)	152.6 (125.8, 181.6	145.6 (121.3, 171.0	0.213
Microorganisms				
*Pseudomonas aeruginosa*	**77 (35.5)**	**58 (45.0)**	**19 (21.6)**	**<0.001**
*Haemophilus influenzae*	34 (15.7)	22 (17.1)	12 (13.6)	0.496
*Nontuberculous mycobacterium*	8 (3.7)	5 (3.9)	3 (3.4)	1.000
*Streptococcus viridans*	3 (1.4)	3 (2.3)	0 (0)	0.273
*Candida albicans*	19 (8.8)	8 (6.2)	11 (12.5)	0.107
*Acinetobacter baumannii*	5 (2.3)	5 (3.9)	0 (0)	0.082
*Neisseria bacteria*	16 (7.4)	6 (4.7)	10 (11.4)	0.063
*Klebsiella pneumoniae*	2 (0.9)	2 (1.6)	0 (0)	0.516
*Enterobacter cloacae*	4 (1.8)	2 (1.6)	2 (2.3)	1.000

Data are presented as mean ± standard deviation, median (interquartile range), or numbers (percentages). BMI: body mass index; CHA: cold hemagglutination; CI: confidence interval; CRP : C-reactive protein; DPB: diffuse panbronchiolitis; ESR: erythrocyte sedimentation rate; FEF: forced expiratory flow; FEV_1_: forced expiratory volume in 1 second; FVC: forced vital capacity; OR: odds ration; P_a_CO_2_: arterial pressure of carbon dioxide; P_a_O_2_: arterial pressure of oxygen; pH: potential of hydrogen; RV: residual volume; WBC: white blood cell. ^※^Disease duration was defined as the time from the first DPB symptoms onset to the first visit to hospital. ^▲^Comparison of DPB subjects with and without bronchiectasis. ^■^A positive CHA test was defined as a value greater than 1 : 64.

**Table 2:** Medications, therapeutic effects, and exacerbation frequencies in DPB subjects with and without bronchiectasis during follow-up.

Treatment and outcome	Whole group (*n*=217)	DPB with Bronchiectasis (*n*=129)	DPB without Bronchiectasis (*n*=88)	*P* value^▲^
Macrolide antibiotics				0.572
Azithromycin	179 (82.5)	104 (80.6)	75 (85.2)	
Clarithromycin	23 (10.6)	15 (11.6)	8 (9.1)	
Erythromycin	12 (5.5)	7 (5.4)	5 (5.7)	
Roxithromycin	3 (1.4)	3 (2.3)	0 (0)	
Regular ICS + LABA	35 (16.1)	18 (14.0)	17 (19.3)	0.291
Regular LAMA	28 (12.9)	18 (14.0)	10 (11.4)	0.576
Follow-up period (months)	32.0 (18.5, 41.0)	35.5 (16.0, 45.0)	29.0 (21.0, 39.0)	0.192
Exacerbation frequency (time/year)^■^	**0.5 (0, 1.5)**	**0.7 (0, 1.6)**	**0.3 (0, 1.3)**	**0.048**
Treatment efficacy				**<0.001**
Healed	36 (16.6)	18 (20.5)	18 (14.0)	
Improved	102 (47.0)	53 (60.2)	49 (38.0)	
Unchanged	39 (18.0)	6 (6.8)	33 (25.6)	
Deteriorating	32 (14.7)	7 (8.0)	25 (19.4)	
Recurrent	8 (3.7)	4 (4.5)	4 (3.1)	
Overall response				**<0.001**
Responsive	138 (63.6)	67 (51.9)	71 (80.7)	
Unresponsive	79 (36.4)	62 (48.1)	17 (19.3)	

Data are presented as median (interquartile range) or numbers (percentages). DPB: diffuse panbronchiolitis; ICS: inhaled corticosteroid; LABA: long-acting bronchial agonist; LAMA: long-acting muscarinic antagonist. ^▲^Comparison of DPB subjects with and without bronchiectasis. ^■^The exacerbation frequency (time/year) was defined as the number of exacerbation events/duration of follow-up (months) × 12.

**Table 3:** Univariate and multiple Cox regression analyses for predicting the time to exacerbation since beginning of macrolide therapy in newly diagnosed DPB patients.

Parameter	Univariate analysis				Multivariate analysis			
	*β* value	Standard error	OR (95% CI)	*P* value	*β* value	Standard error	OR (95% CI)	*P* value
Age	0.010	0.006	1.010 (0.999, 1.021)	0.083	—	—	—	—
Female (yes/no)	−0.029	0.189	0.971 (0.670, 1.407)	0.876	—	—	—	—
Smoking (yes/no)	0.350	0.221	1.419 (0.919, 2.190)	0.114	—	—	—	—
Disease duration (year)	**0.049**	**0.023**	**1.050 (1.004, 1.099)**	**0.034**	—	—	—	—
Chronic sinusitis (yes/no)	0.576	0.333	1.779 (0.927, 3.414)	0.083	—	—	—	—
CHA test (positive/negative)	−0.256	0.263	0.775 (0.462, 1.298)	0.332	—	—	—	—
BMI (kg/m^2^)	**−0.103**	**0.024**	**0.902 (0.861, 0.946)**	**<0.001**	**−0.058**	**0.027**	**0.943 (0.859, 0.994)**	**0.028**
FEV_1_ (% pred)	**−0.028**	**0.007**	**0.972 (0.959, 0.986)**	**<0.001**	**−0.016**	**0.008**	**0.984 (0.969, 1.000)**	**0.045**
*Pseudomonas aeruginosa* culture (positive/negative)	**0.894**	**0.197**	**2.445 (1.660, 3.601)**	**<0.001**	**0.444**	**0.219**	**1.559 (1.014, 2.359)**	**0.043**
Concomitant bronchiectasis (yes/no)	**0.752**	**0.199**	**2.121 (1.437, 3.131)**	**<0.001**	**0.435**	**0.214**	**1.545 (1.016, 2.349)**	**0.042**

BMI: body mass index; CHA: cold hemagglutination; FEV_1_: forced expiratory volume in 1 second; OR: odds ratio; CI: confidence interval.

## Data Availability

The data used to support the findings of this study are included within the article.
